# Supplementary figures and images for: Rest-task modulation of fMRI-derived global signal topography is mediated by transient coactivation patterns
Source: PLoS Biol. 2020 Jul 10;18(7):e3000733. doi: 10.1371/journal.pbio.3000733 (PMC7375654; doi:10.1371/journal.pbio.3000733)

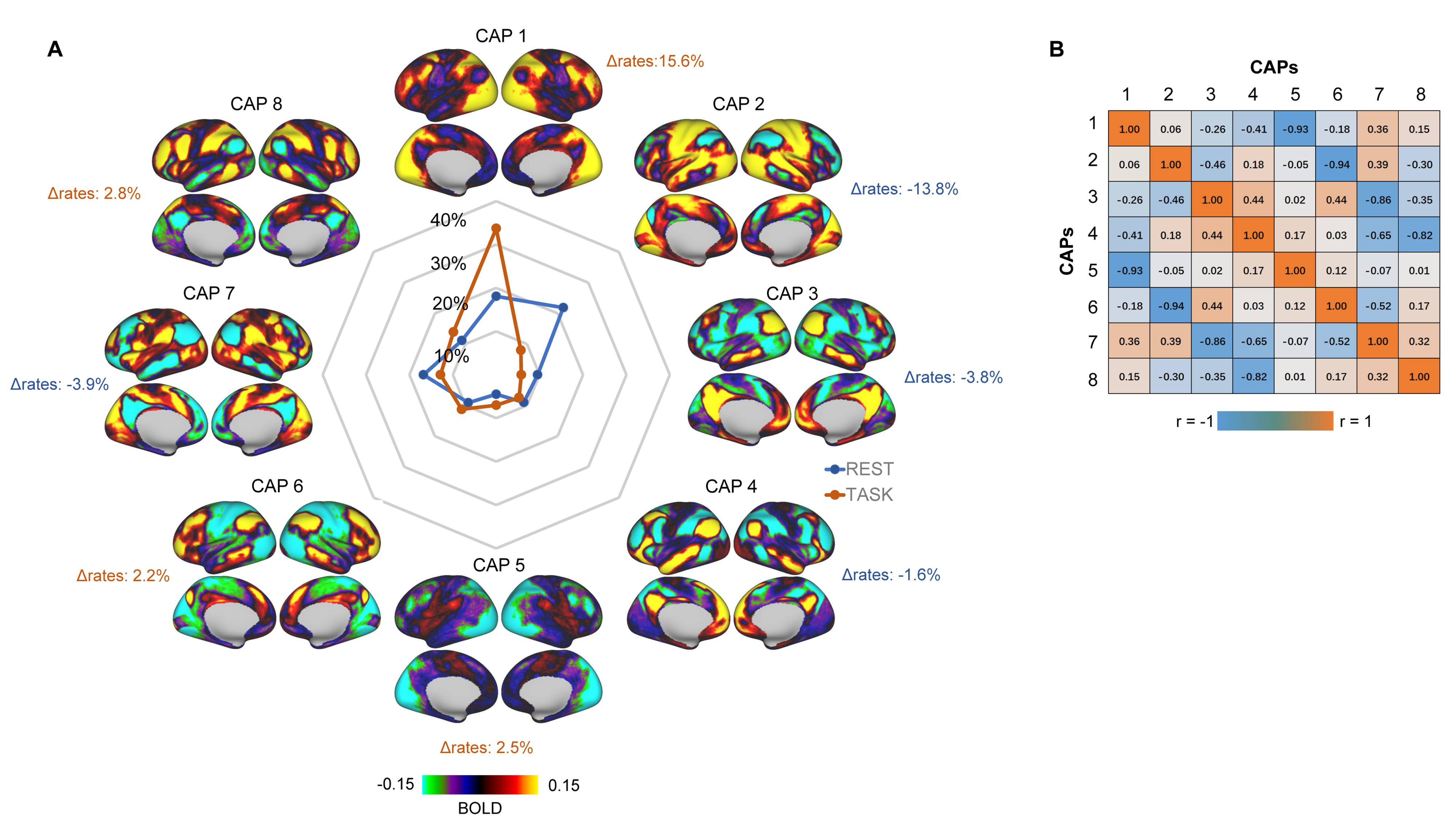

Supplement: S1 Fig — (A) Spatial topography of CAPs and their occurrence rates at the time points of GS-peak. Task modulation denoted the difference in the occurrence rate of the CAPs between task and resting state (Δrate). (B) Spatial correlation between the CAPs. The eight CAPs were composed of four pairs of opposite CAPs (i.e., CAP1 versus CAP5, CAP2 versus CAP6, CAP3 versus CAP7, and CAP4 versus CAP8), as denoted by their negative correlations. Data are available at Dryad: https://doi.org/10.5061/dryad.xsj3tx9bw. CAP, coactivation pattern; GS, global signal. (TIF) [file pbio.3000733.s001.tif]

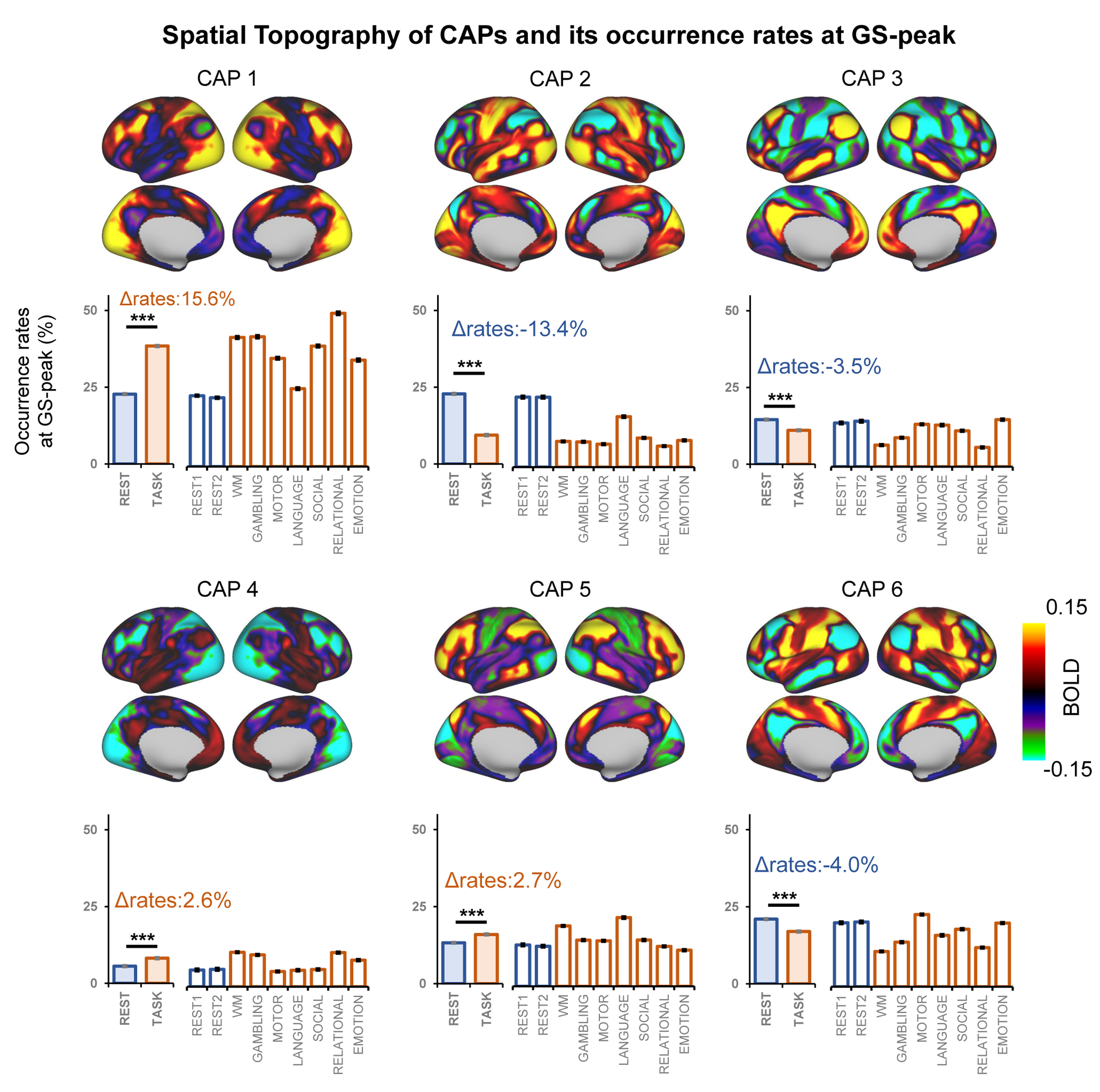

Supplement: S2 Fig — Top panel yielded the CAPs in Fig 3. Bottom panel yielded the occurrence rate across resting state and seven tasks. Data are available at Dryad: https://doi.org/10.5061/dryad.xsj3tx9bw. CAP, coactivation pattern. (TIF) [file pbio.3000733.s002.tif]
