# Supplementary material for: Rest-task modulation of fMRI-derived global signal topography is mediated by transient coactivation patterns
Source: PLoS Biol. 2020 Jul 10;18(7):e3000733. doi: 10.1371/journal.pbio.3000733 (PMC7375654; doi:10.1371/journal.pbio.3000733)
Supplement: Attachment — Submitted filename: rebuttal(20200321).docx [file pbio.3000733.s003.docx]

Reviewer #1:

The authors performed global signal correlation (GSCORR) analysis, and found the pattern is stable across resting-state and tasks. They also found tasks can modulate the GSCORR pattern, and claimed this is reflecting phase coherence rather than amplitude variation.

Major concerns:

1. It has no doubt that GSCORR has a stable pattern, as summarized very well in Power et al.’s 2017 paper (Sources and implications of whole-brain fMRI signals in humans). Power’s paper has a clear demonstration of GSCORR pattern and interpretation of sources of GSCORR, but the viewpoints were not thoroughly discussed (although cited) in the current manuscript. So as this Liu et al.’s 2017 paper: The global signal in fMRI: Nuisance or Information?

**Reply: We thank the reviewer for raising this critical question and we apologize for the insufficient interpretation of the rest-task modulation of global signal and its topography. To probe the question as whether the global signal in fMRI is nuisance or informative, we conducted additional analyses and tried to understand the underlying mechanism of rest-task modulation of global signal topography. We asked that whether global signal topography is dynamic and can be traced to transient co-activation patterns, as inspired by recent studies on co-activation patterns (Liu, de Zwart et al. 2018, Gutierrez-Barragan, Basson et al. 2019, Huang, Zhang et al. 2020). Additionally, as pointed out by Power et al. (2017), the global signal topography is akin to the effects of respiration including its specific topographical distribution, we compared the spatial similarity of the global signal topography with the respiration-based topography (using timeseries of respiration volume per time (RVT)) in both resting state and tasks.**

**In this revised manuscript, we demonstrated that the global signal in fMRI is informative with a strong functional relevance. First, as shown in previous version, the global signal topography showed rest-task modulations, mainly by decreased global signal correlation in somatosensory areas during tasks (Fig. 2). Secondly, our new findings showed that these rest-task modulations can be traced to the dynamics of the global signal as manifested in transient co-activation patterns at the peak period of global signal. These data suggest that 1) global signal topography is constituted by a combination of several co-activation patterns, and 2) rest-task modulation consists in changing the number of occurrences, i.e., occurrence frequency of the co-activation patterns (see Fig. 3 and Fig. 4).**

**Finally, regarding the respiration effect, we observed that the topographical distribution of respiration effects mimicked the topography of the global signal in the resting-state, supporting a close relationship of the two as shown by Power et al. (2017). We extend these findings by showing that, unlike in the resting state, global signal topography during tasks differed from the respiration-based topography. Given the distinct rest-task modulations of the global signal topography and respiration-based topography, we concluded that the global signal (and its neural basis) cannot be fully accounted for by respiration effects (Fig. 5).**

**In sum, we here presented three main findings including (i) rest-task modulation of global signal topography, (ii) the dynamic nature of the global signal with transient co-activation patterns, and (iii) its partial dissociation from respiration-related effects. These findings suggest that global signal topography is not just nuisance but is informative of our brain’s neural activity.**

2. The authors’ main point “the GS topography (GSCORR) exhibited a stable spatial pattern across rest and task, supporting the term as intrinsic architecture” is not innovative. Changing the seed time series from global signal to other seeds (e.g., posterior cingulate cortex for mapping default mode network, or V1 to mapping visual network), will have similar results: stable pattern across tasks but can be modulated by tasks. Even more radically, instead of performing any functional connectivity analysis (correlation analysis), simply averaging all the volumes can have similar effect. However, I don’t think these kind results solved any “fundamental question” (e.g., GS topography is whether it is an intrinsic architecture).

**Reply: We agree with the reviewer that instead of focusing on whether the global signal topography is an intrinsic architecture, it is more important to investigate whether the global signal topography is informative. As mentioned in point 1, we observe that global signal topography is constituted by a dynamic combination of several transient co-activation patterns. As suggested by the reviewer in the first comment, the critical question is whether the global signal is informative; for this reason, we have moved the focus of our paper to investigating the informative nature of the global signal topography. We have changed the title accordingly, that is, ‘Rest-task Modulation of fMRI-derived Global Signal Topography is Mediated by Transient Co-activation Patterns’.**

3. The authors’ conclusion, “phase coherence, rather than amplitude variation, serves as the neural basis of GS architecture”, can not be validated through their analyses. To make such a conclusion, the authors compared global signal Kuramoto change and standard deviation change. However, the calculation of global signal Kuramoto is very similar to global signal correlation by definition, the authors can test these two on simulated time series. In standard deviation calculation, no global signal is involved, thus it’s not surprising that this measure will have results more distant to global signal correlation.

“The contribution to global signal from each grayordinate can depend on either the level of amplitude or the level of phase coherence”. The authors directly used the amplitude (standard deviation) of the signal of a certain grayordinate. However, the more important question is how much the global signal was present in the signal of this grayordinate (i.e., the fraction of the amplitude of GS component present in the signal of this grayordinate).

**Reply: Following the reviewer’s suggestions, we have removed this part of results, and we now focus on the dynamics of rest-task modulation of global signal topography in terms of the underlying transient co-activation patterns.**

**Additionally, regarding the issue about how much the global signal was present in the signal of the grayordinates, we demonstrate that the contribution (measured by global signal correlation (GSCORR)) ranges from around 0 to 0.3 across the grayordinates and varies from rest to tasks (Fig. 1).**

Minor concerns:
1. Multiple comparison correction. For voxel/vertex-wise comparison, the authors “used the threshold at |Cohen’s d| > 0.2 for further analyses (the threshold for reaching small effect size, corresponding to |t-values| > 5.78)”. However, there were so many voxels/vertices across the brain, the authors should perform some kind of multiple comparison correction for this number of voxels/vertices.

**Reply: As suggested, we have performed whole brain FDR-correction in all the grayordinate-based analyses, and used a strict threshold at p<0.001 after FDR correction. The rest-task modulations of global signal topography are robust to such strict threshold.**

2. The authors concluded that GSCORR was not explained by physiological noise by simply compare the results with and without nuisance covariates regression. More methods like in Power et al. 2017 paper should be further tested.

**Reply: This is an excellent suggestion. In this revised manuscript, we have tested the contribution of physiological noise in two aspects. First, regarding the close relationship between the global signal and respiration, as mentioned in Power et al. 2017, we compared the spatial similarity between global signal topography and RVT-based topography (Fig. 5). We found that the relationship between RVT and global signal is state-dependent. Specifically, we observed a high spatial similarity between global signal topography and RVT topography in the resting state, but their spatial similarity was significantly decreased during task states.**

**Second, regarding other physiological noise, we have provided additional control analyses to compare different procedures of noise regression: 1) without any noise regression; 2) regressors including head motion and signals from the ventricle and white matter as well as their first order derivatives; and 3) regressors including those in 2) plus the RVT and heart rate (Fig. 7).**

3. The authors regressed out cardiac and respiratory signals. How did they calculate these two signals?

**Reply: We apologize for the unclear description. In brief, respiration recording was transformed into respiration volume per time (RVT) corresponding to the difference between upper and lower envelope. The cardiac signal was computed in beats-per-minute (bpm) by multiplying the inverse of the time differences between pairs of adjacent peaks with 60. The RVT and heart beat were then resampled to the sampling rate of fMRI (TR = 0.72 s). We have added the detailed description in the revised methods.**

4. Reference style inconsistent. E.g., Line 54: “disorders like schizophrenia (14) and bipolar disorder (Zhang et al. 2018)”.

**Reply: corrected**

5. Line 386. Workbench not Workbranch.

**Reply: corrected**

6. Line 436. Please explain what theta is in this formula.

**Reply: We have removed this part of analysis (i.e., phase coherence vs amplitude variation), as mentioned above.**

Power, J.D., Plitt, M., Laumann, T.O., Martin, A. (2017). Sources and implications of whole-brain fMRI signals in humans. Neuroimage, 146, 609-625, doi:10.1016/j.neuroimage.2016.09.038.

Liu, T.T., Nalci, A., Falahpour, M. (2017). The global signal in fMRI: Nuisance or Information? Neuroimage, 150, 213-229, doi:10.1016/j.neuroimage.2017.02.036.


Reviewer #2:

In this manuscript, Zhang and colleagues comprehensively examine properties of global fMRI signal in a large cohort of adults. They assess the stability of global signal across two resting-state runs, then the spatial topology modulated by tasks. Differences were observed and appropriately quantified by task state, and further analysis revealed that that these differences were likely due to changes in phase coherence (rather than amplitude variation). The authors further argue against a role for the basal forebrain (which I do not find convincing, see below). Overall, the work is rigorous, innovative, timely and compelling.

**Reply: We thank the reviewer for the very positive comments and we are happy to address all the questions.**

2 minor revisions are recommended:

1) A very recent paper provides very complementary findings to the current work: Li et al (2019). Topography and behavioral relevance of the global signal in the human brain. Scientific Reports, 9, 14286. Although highly relevant, the Li paper does not diminish the novelty of the current report. In using the same sample, the global signal topology is again observed (not a true replication). However, the Li paper should be highlighted in the present paper for conveying the intrinsic structure of the global signal (i.e. network architecture underlying the signal) as well as the associations with behavior, thereby providing evidence that the global signal is meaningful. The current manuscript would benefit by including the Li paper in the intro, and relating the findings in the discussion to these observations.

**Reply: Great suggestion. We fully agree with the reviewer that the work by Li et al., 2019 is highly relevant to our study. We have cited this article in the introduction and discussion. Additionally, together with the suggestions by Reviewer #1, we now extend the investigation of the functional relevance of global signal topography by performing additional dynamic analyses, i.e., transient co-activation patterns at the peak of global signal. We have updated the findings by showing that the rest-task modulation of global signal topography can be traced to a set of co-activation patterns at the peak of global signal (Fig. 3 and Fig. 4).**

2) I do not find the basal forebrain findings to be compelling. The authors are essentially interpreting the null hypothesis, which is problematic. The absence of a task > rest difference in basal forebrain contributions to global signal is not evidence for a lack of involvement. The basal forebrain is a very small structure with poor SNR, which may contribute to a Type II error. Further, the type of signals may not be sensitive to broad differences, but as possibly observable in different analytic frameworks, like representation similarity analysis. The authors must be far more conservative in their interpretation of the null effect of basal forebrain.

**Reply: We fully agree with the reviewer’s suggestion and interpretations. We have added one passage in the discussion part as a limitation of our results.**

**“We did not find statistically significant difference of the rest-task modulation in the basal forebrain contributions to global signal. However, caveats need to be taken for interpreting the null effect. That is, the absence of a rest-task difference could not be evidence for a lack of involvement of the basal forebrain. First, the basal forebrain is a very small structure with poor SNR (Liu et al., 2018), which may contribute to a Type II error. Second, our analytical approach may be not sensitive enough to detect the basal forebrain’s contribution under the broad differences between resting and task states.”**

**Nevertheless, from another angle, our results support the findings by Liu et al., 2018, in terms of the strong negative correlation between the basal forebrain and global signal in general. We found that the negative correlation is robust across both the resting state and task states,. The seemingly invariant negative correlation may indicate that the arousal level is similar across the resting and task states, and rest-task modulation most likely occurred on the cortical level that is associated with cognitive processing.**


Reviewer #3:

The novelty and significance: The first exploration of an intrinsic architecture of global signal in the human brain.

Technical merit and the experimental design: HCP data is basing the technical advances on the most benefited neuroimaging methods. The whole experimental design is discovery and exploratory.

Sufficiency of the statistical analysis: There lacks a set of comprehensive statistical assessments on the global brain signal either for phase or amplitude. It seems a huge mount of spatial correlations done thought the whole analyses, however such analyses are problematic where the huge number of voxels (samples) are highly correlated in terms of their spatial adjacency. It is better to figure out a novel way to qualify the spatial similarity of the brain mapping measurements across different states of tasks and rests. One possibility would be Kendall's statistics (Jiang & Zuo, 2016, The Neuroscientist). It would be also another way to strengthen the statistical and translational significance by performing a test-retest reliability of the measurements on the global signal with the HCP test-retest data (please read Zuo et al., 2019, Nat Hum Behave for a reference).

**Reply: We appreciate the reviewer’s suggestions. We have removed the part of phase vs. amplitude analysis, as it seems to be a trivial comparison when the fMRI signal is normalized into z-scores.**

**To minimize the potential that the local spatial adjacency may lead to a high spatial similarity, we have performed the spatial similarity analysis at the region-of-interest level based on a parcellation with 1000 ROIs (Schaefer et. al., 2018). In addition, we examined the test-retest reliability for the resting state data acquired at two different days. We observed almost identical global signal topography for the two resting states with a high spatial similarity between the two (r=0.99).**

All data needed to replicate the study: The HCP data is publicly shared to the community. Regarding the observations reported, the authors need to add some analyses of their reproducibility such as a replication with another public database (e.g., CoRR or INDI Lifespan Data).

**Reply: We fully agree on the importance of replication. We have now used an independent 7 Tesla dataset from the HCP including resting state and two different tasks (i.e., movie watching and retinotopy stimuli processing). The main findings with respect to the rest-task modulation of global signal topography have been replicated (Fig. 8).**
