# Supplementary material for: Rest-task modulation of fMRI-derived global signal topography is mediated by transient coactivation patterns
Source: PLoS Biol. 2020 Jul 10;18(7):e3000733. doi: 10.1371/journal.pbio.3000733 (PMC7375654; doi:10.1371/journal.pbio.3000733)
Supplement: Attachment — Submitted filename: reply to AE comments.docx [file pbio.3000733.s004.docx]

Dear Roli

I’m afraid the additional analysis does not account for the confound I noted on the last round. As a result the ‘caveats’ offered as additional paragraphs by the authors are not strong enough. They note the confound I indicated; but then they propose that their analysis in Figs 3,4 and 5 make my suggested confound unlikely and therefore not an issue of concern. That is not the case.

As I see it the problem arises from the fact that the ‘resting state’ has (trivially) no task and no temporal structure. While each of the task states trivially does. This task structure imposes temporal structure on both the GS (due to task-linked breathing and HR changes) and task-linked BOLD responses (e.g. from visual or other stimuli associated with the task, responses such as button presses, which are necessarly entrained to the task); both GS and task-linked BOLD would have significant components along the vector defined by the response time course, making them hard to separate. Simply comparing estimates of GS during task vs rest would be confounded by this difference in temporal structure.

It is striking that the biggest difference between the resting state GS and the overall task GS consists of reduced activity in the somatomotor and visual region, as well as the default mode network (DMN). Fig 3B (see detail below). The reduced activity in the somatomotor region and DMN are clear as they are spatially distinct from their surrounds. The reduced visual component can be appreciated by comparing with the corresponding region in the resting state map. Note that these patterns of reduced activity are consistent with the changes shown (very nicely) by the authors in their Fig 4B

Consider the following, consistent with my null hypothesis that the GS is unchanged during the task:

1: During the task, breathing and HR get entrained to task timing. Thus the task GS has a temporal structure which is not there at rest even though it maintains a fixed spatial pattern over the brain.

2: The measured BOLD is the sum of the GS and task-linked visual stimulation, somatomotor activity (manual button presses, etc) cognitive responses, all entrained to the same task timing.

3: Due to the dynamics of the BOLD response, the contributions from respiration + HR as vs visual / somatomotor / cognitive could have different, possibly opposite phases relative to task timing.

The task-linked BOLD responses would then have a significant projection along the same (temporal) vector as the GS, but opposite in sign. This could, in effect, reduce the apparent strength of the GS over the somatomotor and visual regions of cortex. The task would also lead to an antiphase DMN.

The authors’ Fig 5 does not offer strong evidence to the contrary. The dynamic range of the maps in Fig 5B (0 – 0.05) is about an order of magnitude lower than in Fig 4B shown above. The small map differences shown in Fig 5B are thus hard to interpret.

Because the BOLD signal due to entrained physiology and due to task-linked neural activity are unavoidably correlated in time, I’m not sure they can be easily separated. The analysis offered by the authors does not eliminate the null hypothesis. I believe their caveat should reflect this more clearly.

**Reply:**

We appreciate the editor’s very thoughtful comments and apologize for our misinterpretation in last reply. The null hypothesis and related reasoning were very well taken. While we fully agree to take the editor’s point of view as an alternative interpretation of our results (reflected in the edited discussion part), we do have a few considerations to share.

1. It may be theoretically and experimentally rather difficult to dissociate the ‘real’ global signal from task-evoked activity. Specifically, for testing whether the rest-task modulation of global signal is a result of phase entrainment to task timing, one approach is to first identify the task-evoked activity. The common way is using GLM model. However, it is hard to ascertain if the residual signal is task-irrelevant. For instance, we and another group both demonstrated that resting-state activity and task-evoked activity negatively interact with each other, and task evoked activity is modulated by pre-stimuli activity, suggesting that theoretically, GLM alone is unable to totally explain the task-evoked activity by itself independent of potential non-linear carry-over effects from spontaneous activity, i.e., prestimulus and/or resting state (He, 2013; Huang et al., 2017).
2. Another approach may be to first identify the ‘real’ global signal, and its projection on gray matter. However, the ‘real’ global signal in terms of its neural or physiological sources remain unclear. The potential candidates could be the basal forebrain as its neural source (Liu et al., 2018; Turchi et al., 2018) and respiration as its physiological source (Power, Plitt, Laumann, & Martin, 2017). However, both of them only partially correlate with the global signal (as demonstrated in our data). It is noteworthy that, the resting-state global signal can be considered a superposition of the global signal projection from basal forebrain, physiological signal (including respiration), and the overall activity of co-activation patterns (CAPs) during specific phases of the global signal. As reported by Gutierrez-Barragan, Basson, Panzeri, and Gozzi (2019), the reoccurring CAPs are phase locked to the global signal (see the graphical abstract of their study below). Nevertheless, we completely agree that more investigations are needed to uncover the origin of the global signal and the spatial co-activation pattern entrained to it.

3. While one could take either a static (time-averaged) view or a dynamic view (transient co-activation pattern) on the global signal, we deem that the latter may have more explanatory power. For example, it may meet the observations of altered global signal topography in psychiatric disorders, when no task entrainment effect is involved. Specifically, the spatial pattern of our identified CAPs resembles the global signal topography change in schizophrenia (Yang et al., 2017) (see the figure for spatial comparison below). This may suggest that abnormal global signal topography in psychiatric disorders can be indexed by the occurrence rate of reoccurring spatial co-activation patterns.

4. We also appreciate the editor’s comment on the task-unspecific changes especially the reduction in somatosensory cortex. We argue that this reduction may not be the result of task-related anticorrelation. During the motor task, which is highly relevant to motor behavior, it did not show the highest anticorrelation (see the figure below). In contrast, the decrease was higher during other non-motor tasks – we therefore assume that these reductions are task-unspecific decreases. This, is in part, based on the observations that the somatomotor cortex has a high contribution to the global signal during resting state, and that the global signal correlates with respiration in a higher level during rest than task. One possible interpretation could be that the contribution of somatomotor cortex may be related to physiological-related internal processing (e.g., respiration and heart beat); its task-related decrease may then indicate that global signal is modulated more by external stimuli through the increased occurrence rate of visual CAP and/or phase entrainment to task timing (with the latter being suggested by the academic editor).

We have now revised our discussion to make the caveat clearer, according to the editor’s comments.

*Although we demonstrated that the rest-task modulation of global signal was associated with alterations of the prevalence of CAPs, an alternative interpretation (or a potential confound) of the results may be considered. More specifically, the ‘resting state’ has (trivially) no task and no temporal structure, whereas each of the task states trivially does. This task structure may impose temporal structure on both the GS (due to task-linked breathing and HR changes) and task-linked BOLD responses (e.g. from visual or other stimuli associated with the task, responses such as button presses, which are necessarily entrained to the task); both GS and task-linked BOLD would have significant components along the vector defined by the response time course, making them hard if not impossible to separate. Simply comparing estimates of GS during task vs rest would be confounded by this difference in temporal structure of rest and task states. Due to the fact that within the BOLD signal both entrainment to the temporal structure of the task and task-linked neural activity are unavoidably correlated in time, it remains methodologically rather challenging to decompose these two components in a meaningful way.*

**Reference**

Gutierrez-Barragan, D., Basson, M. A., Panzeri, S., & Gozzi, A. (2019). Infraslow State Fluctuations Govern Spontaneous fMRI Network Dynamics. *Curr Biol, 29*(14), 2295-2306 e2295. doi:10.1016/j.cub.2019.06.017

He, B. J. (2013). Spontaneous and task-evoked brain activity negatively interact. *J Neurosci, 33*(11), 4672-4682. doi:10.1523/JNEUROSCI.2922-12.2013

Huang, Z., Zhang, J., Longtin, A., Dumont, G., Duncan, N. W., Pokorny, J., . . . Weng, X. (2017). Is there a nonadditive interaction between spontaneous and evoked activity? Phase-dependence and its relation to the temporal structure of scale-free brain activity. *Cerebral Cortex, 27*(2), 1037-1059.

Liu, X., de Zwart, J. A., Scholvinck, M. L., Chang, C., Ye, F. Q., Leopold, D. A., & Duyn, J. H. (2018). Subcortical evidence for a contribution of arousal to fMRI studies of brain activity. *Nat Commun, 9*(1), 395. doi:10.1038/s41467-017-02815-3

Power, J. D., Plitt, M., Laumann, T. O., & Martin, A. (2017). Sources and implications of whole-brain fMRI signals in humans. *Neuroimage, 146*, 609-625. doi:10.1016/j.neuroimage.2016.09.038

Turchi, J., Chang, C., Ye, F. Q., Russ, B. E., Yu, D. K., Cortes, C. R., . . . Leopold, D. A. (2018). The Basal Forebrain Regulates Global Resting-State fMRI Fluctuations. *Neuron, 97*(4), 940-952 e944. doi:10.1016/j.neuron.2018.01.032

Yang, G. J., Murray, J. D., Glasser, M., Pearlson, G. D., Krystal, J. H., Schleifer, C., . . . Anticevic, A. (2017). Altered Global Signal Topography in Schizophrenia. *Cereb Cortex, 27*(11), 5156-5169. doi:10.1093/cercor/bhw297
